# Effect of Porcine- and Bovine-Derived Xenografts with Hydroxypropyl Methylcellulose for Bone Formation in Rabbit Calvaria Defects

**DOI:** 10.3390/ma16051850

**Published:** 2023-02-23

**Authors:** Su-Hyun Hwang, Keumok Moon, Wen Du, Won-Tak Cho, Jung-Bo Huh, Eun-Bin Bae

**Affiliations:** 1 Department of Prosthodontics, Dental Research Institute, Dental and Life Sciences Institute, Education and Research Team for Life Science on Dentistry, School of Dentistry, Pusan National University, Yangsan 50612, Republic of Korea; 2 Department of Integrated Biological Science, Pusan National University, Busan 46241, Republic of Korea; 3 State Key Laboratory of Oral Diseases, & National Clinical Research Center for Oral Diseases, Department of Prosthodontics, West China Hospital of Stomatology, Sichuan University, Chengdu 610093, China; 4 The Shapiro Family Laboratory of Viral Oncology and Aging Research, Section of Restorative Dentistry, UCLA School of Dentistry, Los Angeles, CA 90095, USA

**Keywords:** bone regeneration, hydrogel, hydroxypropyl methylcellulose, moldability, xenografts

## Abstract

In this study, hydroxypropyl methylcellulose (HPMC) was mixed with particle-type xenografts, derived from two different species (bovine and porcine), to increase the manipulability of bone grafts and compare the bone regeneration ability. Four circular defects with a diameter of 6 mm were formed on each rabbit calvaria, and the defects were randomly divided into three groups: no treatment (control group), HPMC-mixed bovine xenograft (Bo-Hy group), and HPMC-mixed porcine xenograft (Po-Hy group). At eight weeks, micro-computed tomography (µCT) scanning and histomorphometric analyses were performed to evaluate new bone formation within the defects. The results revealed that the defects treated with the Bo-Hy and the Po-Hy showed higher bone regeneration than the control group (*p* < 0.05), while there was no significant difference between the two xenograft groups (*p* > 0.05). Within the limitations of the present study, there was no difference in new bone formation between porcine and bovine xenografts with HPMC, and bone graft material was easily moldable with the desired shape during surgery. Therefore, the moldable porcine-derived xenograft with HPMC used in this study could be a promising substitute for the currently used bone grafts as it exhibits good bone regeneration ability for bony defects.

## 1. Introduction

Bone grafting is a common dental procedure for implant surgery as there is the necessity of increasing the quantity and quality of bones around implants [1]. The primary goal of bone grafting is to restore the form and function of the original bone by filling the defects with bone graft materials [2]. Bone graft materials used in bone grafting are divided into various types, such as autografts, allografts, synthetic grafts, and xenografts [3]. Among the types of bone graft materials, xenografts can typically be of bovine, porcine, or equine origin and can be mass-produced in large quantities at relatively affordable processing costs [4].

Bovine-derived xenograft material has a hydroxyapatite structure and has been used in many surgeries, such as alveolar ridge augmentation, sinus floor augmentation, and bone defect reconstruction [5,6]. Recently, the use of porcine-derived xenografts is increasing, and several studies have reported excellent results, similar to those of bovine-derived xenografts in terms of biocompatibility and bone regeneration [7,8,9,10]. The Ca/P ratio of porcine-derived xenografts is similar to the Ca/P ratio of human bone [11]. In addition, the bone strength of porcine bones is about 200 ± 300 MPa, which is close to 50 ± 389 Mpa of human bone strength [12]. Both xenografts are biocompatible and osteoconductive, meaning that they can be used as bone substitutes without interfering with normal reparative bone processes [13].

The bone particles of particle-type bone grafts lack bonding strength, which leads to poor manipulability. This could result in the bone graft materials being mislocated or lost during graft filling [4,14]. Furthermore, particle-type bone grafts cannot perfectly maintain the space, and their bone quality and shape prognosis are inferior to block-type bone grafts in cases of irregular or significant bone defects [15]. Therefore, block-type bone grafts have been introduced to solve these problems [16,17]. Block-type bone grafts have excellent mechanical strength and shape retention, so they are favorable to be used in relatively large bone defects [18,19]. However, block-type bone grafts require longer healing periods and complicated techniques due to delayed re-vascularization [20].

Recently, many studies have been dedicated to finding a hydrogel that improves bone regeneration and moldability for particle-type bone grafts by using various organic materials, such as hyaluronic acid, collagen, or hydroxypropyl methylcellulose (HPMC) [21,22,23]. These organic materials are used as carrier materials to improve the handling of bone graft powder [24]. In particular, cellulose ethers such as HPMC and methylcellulose have excellent biocompatibility and improve injectability, cohesiveness, and fracture toughness, even in small amounts [25].

HPMC is a cellulose derivative and has been widely used in pills and ophthalmic lenses [26,27], while also demonstrating biodegradability, hydrophilicity, and expansion characteristics in wet conditions [28]. In addition, HPMC has a wet-swell network structure which increases bone formation and provides a nutritional environment suitable for endogenous cell growth [29]. In the previous study [30], bovine-derived xenografts with HPMC did not show cytotoxic effects and exhibited a positive effect on osteoblast differentiation. As a result of the evaluation of bone formation using rat calvaria, bovine-derived xenografts with HPMC obtained similar results compared to the commercially available bovine bone grafts. The HPMC cross-linked keratin scaffold containing HA showed potential application for bone tissue with high cell viability, adhesion, and affinity to proliferate [31]. In another prior study, HPMC with calcium sulfate-based bone graft putty has handling characteristics and the ability to maintain the position within defects [32]. HPMC combined with biphasic calcium phosphate (BCP) also demonstrated improved bone regeneration of tooth extraction sockets [33]. This polymer hydrogel exhibits significant biocompatibility and has been added to bone graft manufacture to increase the manipulability of particulate bone graft materials. Furthermore, several studies have shown that hydrogels are effective mediators of cell delivery [34,35]. Based on these characteristics, the addition of hydrogel to bone grafts increases the viscosity of the bone graft material and improves the bone formability [36].

In this study, HPMC was applied to the porcine-derived xenograft bone, referencing a previous study [30]. There was no research about the bone regeneration capacity of the porcine bone graft combined with HPMC. The purpose of this study was to compare and evaluate the bone regeneration capacity of particle-type xenografts, which are derived from two different species (bovine and porcine) in rabbit calvaria by adding HPMC to increase manipulability.

## 2. Materials and Methods

### 2.1. Experimental Xenogeneic Materials

In this study, two types of xenograft materials were compared. A commercially available bovine-derived xenograft with hydrogel (S1^®^, Medpark, Busan, Republic of Korea) was used (Bo-Hy group). The porcine xenograft material with hydrogel (Po-Hy group) was prepared with the same method which is used to produce the commercially available bovine-derived xenograft with hydrogel. HPMC was added to the commercialized porcine bone graft (BOSS^®^, Medpark, Busan, Republic of Korea), which originally does not include HPMC. The manufactured graft materials were not disclosed in detail by the company [30].

### 2.2. In Vitro Study

#### 2.2.1. Observation of Surface Morphologies

A small amount of each xenograft sample was attached to the mount using carbon tape. The samples were sputter-coated with a 2 nm-thick layer of Pt using a sputtering apparatus (Q150T ES, Quorum Technologies, East Sussex, UK), resulting in a highly conductive coating that allowed for improved imaging. Scanning electron microscopy was performed to compare the surface morphology of bovine and porcine xenogeneic bone materials at magnifications of ×125, ×500, ×3000, and ×10,000. Samples were imaged on a scanning electron microscope (FE-SEM, Zeiss Gemini 500, ZEISS, Oberkochen, Germany).

#### 2.2.2. Chemical Composition

Energy-dispersive X-ray spectrometry (EDX, Oxford Link ISIS 300, Oxford, UK) was used to estimate the relative abundance of sample surface elements. A quantitative analysis was performed by taking distinct sites of interest from both the center and periphery of samples by three random points. The concentration of a certain element contained in a sample was measured at a voltage of 15 kV. The target elements were carbon (C), oxygen (O), phosphorus (P), and calcium (Ca), which are the surface components of the porcine bone graft materials [37].

#### 2.2.3. Preparation of Extracts for In Vitro Cell Assay

The xenograft extracts for the in vitro cell assay were prepared according to the method described by Bae et al. [38]. In brief, 1 g of each xenograft was mixed with 10 mL of alpha-modification of Eagle’s medium (α-MEM; Welgene, Daegu, Republic of Korea) and stored at 37 °C under 5% CO_2_ in a humidified culture chamber for 24 h. The medium was separated from the xenografts by centrifugation at 1200× *g* for 5 min, filtered through a membrane (0.2 μm), and stored at 4 °C until use. The xenograft extracts were mixed with cell culture medium in a ratio of 1:4 (*v*/*v*) for the in vitro cell assay.

#### 2.2.4. Cell Culture Conditions

MC3T3-E1 cells (subclone 4, ATCC, Manassas, VA, USA) were cultured in α-MEM supplemented with 10% fetal bovine serum (FBS, Gibco, Waltham, MA, USA) and 1% antibiotics (penicillin 10,000 U/mL and streptomycin 10,000 mg/mL, Gibco) and maintained at 37 °C under 5% CO_2_ in a humidified culture chamber [39,40]. The culture medium was regularly changed, every three days. For the cell proliferation assay, the cells were cultured in α-MEM containing xenograft extracts, 10% FBS, and 1% antibiotics. For the alkaline phosphatase (ALP) staining and activity assay, and quantitative real-time polymerase chain reaction (qPCR) analysis, the cells were cultured in osteogenic medium (α-MEM containing xenograft extracts, 10% FBS, 1% antibiotics, 50 µg/mL ascorbic acid, and 10 mM β-glycerophosphate).

#### 2.2.5. Cell Proliferation Assay

The cells were seeded into a 96-well cell culture plate (6 × 10^3^ cells/well) and incubated for 1, 3, 6, and 9 days. After incubation, CCK-8 assay solution (Dojindo, Rockville, MD, USA) was added into each well and then incubated for an additional 1 h. One hundred microliters of the medium were transferred to a 96-well plate and absorbance was measured at 450 nm [30].

#### 2.2.6. Observation of Cell Attachment

In order to investigate cell attachments to different xenograft materials, 10 mg of the xenograft was placed into a 48-well cell culture plate and immersed in α-MEM supplemented with 10% FBS and 1% antibiotics for 3 h to prevent the bone graft materials from floating [41]. The cells were loaded into the plate (1.5 × 10^4^ cells/well) and incubated for 7 and 14 days. After incubation, the xenografts were washed three times with phosphate-buffered saline (PBS), fixed in 4% formaldehyde (Sigma-Aldrich, St. Louis, MO, USA) for 2 h, and washed five times with PBS. The xenografts were dehydrated with an increasing ethanol concentration (30%, 50%, 70%, 90%, 90%, 95%, 100%, 100%, 100% for 10 min, respectively) and dried using hexamethyldisilazane (Sigma-Aldrich, St. Louis, MO, USA). Cell attachment to the xenografts was imaged with SEM, as described in Section 2.2.1.

#### 2.2.7. ALP Staining and Activity Assay

The cells were plated into a 12-well plate and cultured for 3, 6, and 9 days in the osteogenic medium for the purpose of evaluating their osteogenic differentiation. ALP staining and activity were assessed according to the method described by Moon et al. [42].

#### 2.2.8. qPCR Analysis

For measurement of mRNA expression in MC3T3-E1 cells toward the grafts, the cells were incubated for 3, 6, and 9 days. qPCR was performed according to the method described by Moon et al. [42]. All reactions were performed in triplicate and the primer sequences are shown in Table 1.

### 2.3. In Vivo Study

#### 2.3.1. Operative Procedures

**Figure 1:**
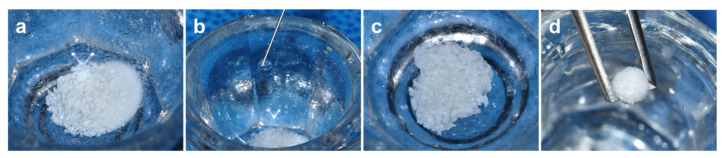
Bone graft material gelation process. (**a**) Prepared 0.25 g of xenograft material, (**b**) added 0.35 cc of saline, (**c**) mixed xenograft material and saline, and (**d**) moldable gelled xenograft material.

**Figure 2:**
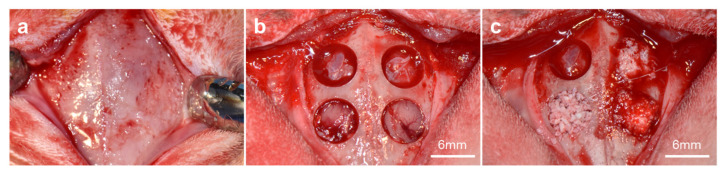
Surgical procedures using a critical-sized calvaria bone defect model in rabbit. (**a**) Exposed rabbit calvarium, (**b**) created four circular defects with a trephine bur, and (**c**) implanted bone materials into the defect area (one defect area assigned as a control group).

The housing and experimental protocols used in this study were approved by the Institutional Animal Care and Use Committee of Chung Buk National University (CBNUA-157R-20-01). Four healthy rabbits (New Zealand white rabbit, 12-week-old, 3.0–3.5 kg) were housed in a temperature-, humidity-, and light-controlled environment and were fed commercial feed pellets and water. The rabbits were administered general anesthesia by intramuscular injection of Tiletamine/Zolazepam (Zoletil^®^, Virbac Korea, Seoul, Republic of Korea) 10 mg/kg, and 1~2% isoflurane (Ifran Liq, Hana Pharm, Seoul, Republic of Korea). The rabbit calvarium region was shaved and disinfected using povidone-iodine. Afterward, 2% lidocaine (Lidocaine HCl, Huons, Seoul, Republic of Korea) with epinephrine 1:100,000 was injected for local anesthesia. Subsequently, the skin was incised along the midline of the frontal bone, and the periosteum was elevated to expose the calvaria. Four circular calvaria defects (diameter, 6 mm) were prepared using a trephine bur (3I Implant innovations Inc., Palm Beach Garden, FL, USA) with continuous saline cooling. The quantified experimental bone grafts (0.25 g) were applied with 0.35 cc of saline to prepare a moldable gelled bone graft lump (Figure 1). The defects were filled with either the Bo-Hy or Po-Hy groups (*n* = 6). The other defects were not assigned with any graft material to compare the efficacy of bone regeneration (Figure 2). The periosteum was repositioned and sutured using 4-0 absorbable vicryl (Ethicon, Somerville, NJ, USA), and then skin was closed using 3-0 non-absorbable black silk (Ailee Co., Seoul, Republic of Korea).

#### 2.3.2. Sacrifice

At 8 weeks after the operation, the rabbits were euthanized by CO_2_ asphyxiation. The calvaria samples were harvested using a diamond disk (Microsaw, Friadent, Mannheim, Germany) on a dental drill unit after incision of the overlying soft tissue. Samples were fixed with 10% buffered formalin for 7 days.

#### 2.3.3. Micro-Computed Tomography (µCT) Analysis

**Figure 3:**
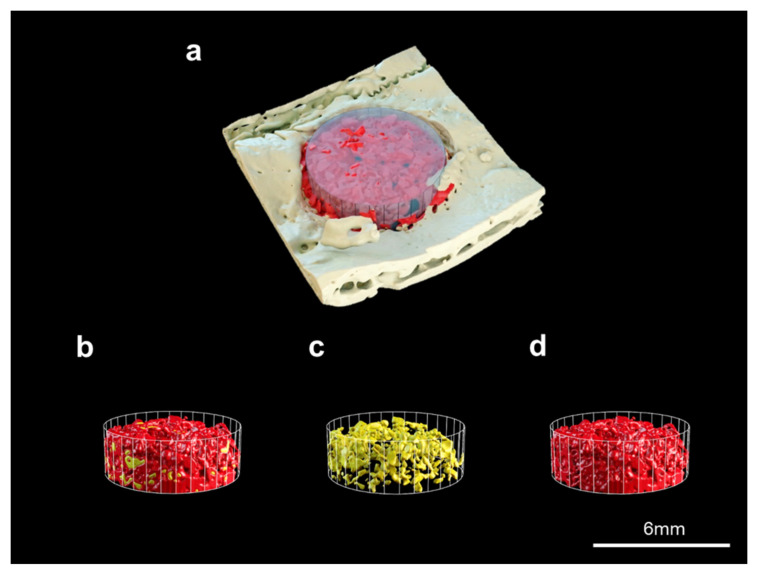
Three-dimensional images of bone volume samples. (**a**) Reconstruction of µCT data, (**b**) separation of region of interest (ROI), (**c**) bone graft materials, and (**d**) new bones (scale bars = 6 mm).

To evaluate the new bone volume of each xenograft site, all the samples were scanned using a µCT (Skyscan-1173, Bruker-CT, Kontich, Belgium) at 130 kV, 60 µm intensity, and 18 µm image resolution. A reconstruction software was used to calculate the three-dimensional (3D) new bone volumes (NBV, mm^3^) of the scaffold (Nrecon ver. 1.6.10.1, Bruker, Kontich, Belgium). To precisely determine the exterior shape of the 3D model, the image-segmented 3D model of the bone scaffold was transformed into STL format. The converted file was imported and rendered using 3D-processing software (Blender Foundation, BlenderTM, Amsterdam, Netherlands). The regions of interest (ROI) were generated as 6 mm in diameter and 2 mm-thick (Figure 3).

#### 2.3.4. Histologic Analysis

**Figure 4:**
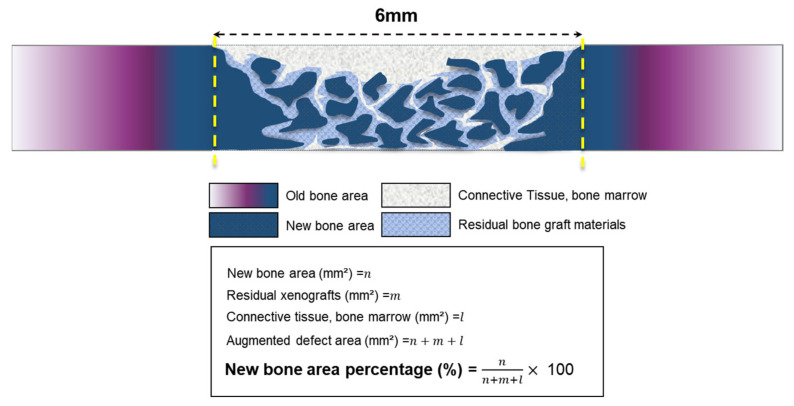
Schematic diagram of histometric analysis.

Samples were decalcified with Calci-Clear^TM^ Rapid (National Diagnostics, 305 Patton Drive, Atlanta, GA, USA) after 7 days of fixation. The sacrificed samples were then dehydrated in alcohol rinses and embedded in paraffin. Embedded specimens were sectioned to a thickness of 4 µm with a microtome (Leica RM2255, Leica Microsystems, IL, USA). The histological slides were stained with hematoxylin and eosin (H&E) and Masson’s trichrome (MT). Images were captured using an optical microscope (BX51, OLYMPUS, Tokyo, Japan) with a charged-coupled device (CCD) camera (Polaroid DMC2 digital Microscope Camera, Polaroi, Cambridge, MA, USA) at ×12.5, ×40, and ×100 magnifications to evaluate the histomorphometry. Captured images were analyzed using i-Solution software (IMT, Daejeon, Republic of Korea) and the percentage of newly formed bone area was consistently measured by one investigator. The parameter measurements are shown in Figure 4.

### 2.4. Statistical Analysis

A one-way ANOVA and Tukey’s post hoc test were used for the comparison of the in vitro results (SPSS ver 25.0, Chicago, IL, USA). To compare the in vivo results, the Kruskal–Wallis test was performed, followed by the Mann–Whitney U post hoc test (Prizm 9, GraphPad, San Diego, CA, USA). Statistical significance was accepted for *p* < 0.05 in all the statistical analyses.

## 3. Results

### 3.1. In Vitro Findings

#### 3.1.1. Scanning Electron Microscope Surface Analysis

**Figure 5:**
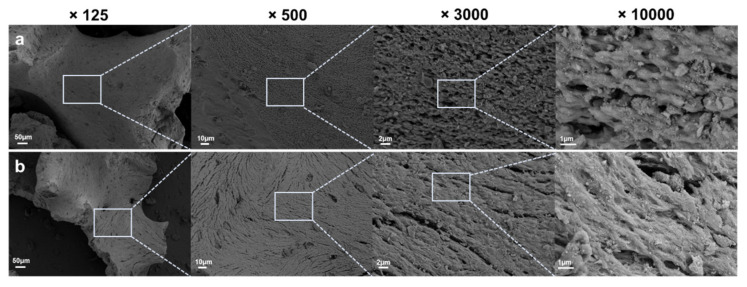
Comparative SEM images of experimental xenografts. (**a**) Bo-Hy and (**b**) Po-Hy. The figure clearly demonstrates macro-porous characteristics and comparable structures of two xenografts.

Surface images of the two xenografts are shown in Figure 5. SEM revealed that the xenograft bone particle properties between the two groups were broadly similar. Both groups showed rough surfaces and similar macro-porous characteristics were observed at magnifications of ×125, ×500, ×3000, and ×10,000.

#### 3.1.2. Energy-Dispersive X-ray Spectroscopy (EDX) Findings

For quantitative analysis of the surface elements of the graft material through EDX analysis, the samples were each measured three times and the average value was calculated. The ratio of elements calcium and phosphorus (Ca/P) was 2.16% in the Bo-Hy group and 2.22% in the Po-Hy group. The elements which had the highest percentages were O, C, Ca, and P (Table 2).

#### 3.1.3. Measurement of Cell Proliferation

**Figure 6:**
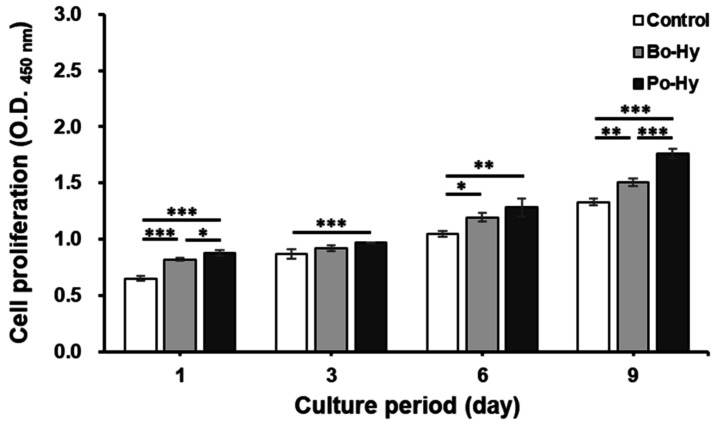
Cell proliferation of xenograft extracts on MC3T3-E1. Significant differences were observed when compared with the controls: * *p* < 0.05, ** *p* < 0.01, *** *p* < 0.001, *n* = 5.

To determine the effect of xenograft extracts on the proliferation of MC3T3-E1 cells, the cells were cultured for 9 days, and the proliferation was assessed using a CCK-8 assay kit (Figure 6). The cell proliferation of the xenograft extracts was slightly increased compared to the control. The cell proliferation rate by Bo-Hy was 126 ± 2%, 106 ± 3%, 114 ± 3%, and 113 ± 3% at 1, 3, 6, and 9 days, respectively, compared to the control. The cell proliferation rate by Po-Hy was 135 ± 4%, 111 ± 1%, 123 ± 8%, and 132 ± 3% at 1, 3, 6, and 9 days, respectively, compared to the control. Porcine- and bovine-derived bone substitutes have shown their suitability as bone graft materials through several studies [38]. The results of this study also showed that both xenografts are non-cytotoxic and effective in cell proliferation.

#### 3.1.4. Cell Attachment

**Figure 7:**
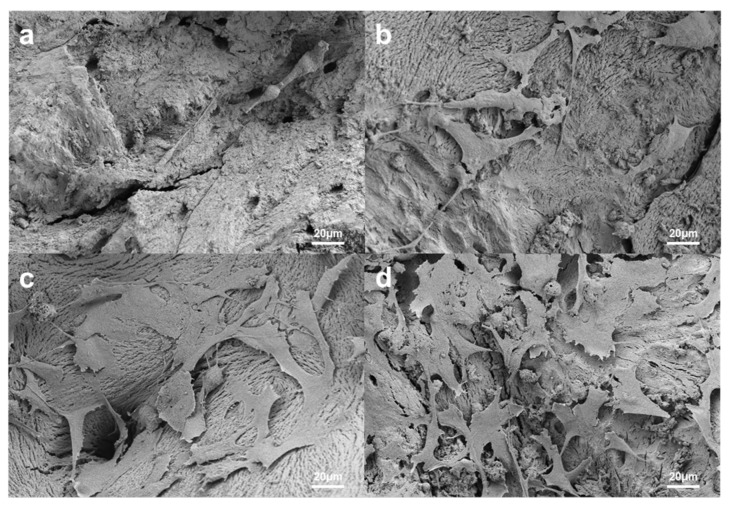
SEM photographs of xenograft surfaces after culture with MC3T3-E1 for 7 and 14 days. (**a**) Bo-Hy, 7 days, (**b**) Po-Hy, 7 days, (**c**) Bo-Hy, 14 days, and (**d**) Po-Hy, 14 days (original magnification ×500).

MC3T3-E1 cells were cultured for 7 and 14 days and observed via SEM to realize the cell attachment profiles of Bo-Hy and Po-Hy (Figure 7). The cells adhered well to the surface of both xenografts and elongated. Similar to the results of the cell proliferation assay, more cells were attached to the surface of Po-Hy than Bo-Hy.

#### 3.1.5. Measurement of ALP Staining and Activity

**Figure 8:**
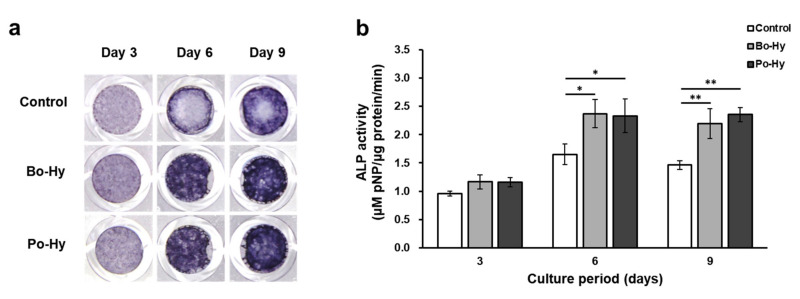
Cell osteogenic differentiation assay. (**a**) ALP staining and (**b**) ALP activity. Significant differences were observed when compared with the controls: * *p* < 0.05, ** *p* < 0.01, *n* = 3.

The activity and staining of ALP, an early marker of osteoblast differentiation, were measured to investigate the effect of the xenograft extract on MC3T3-E1 cells. The ALP staining and activity of xenograft extracts were higher than those of the control and there was no significant difference between the Bo-Hy and Po-Hy extracts (Figure 8). The ALP activities of both xenografts were approximately 20%, 40%, and 50% higher on days 3, 6, and 9, respectively, compared to the controls.

#### 3.1.6. Analysis of qPCR

**Figure 9:**
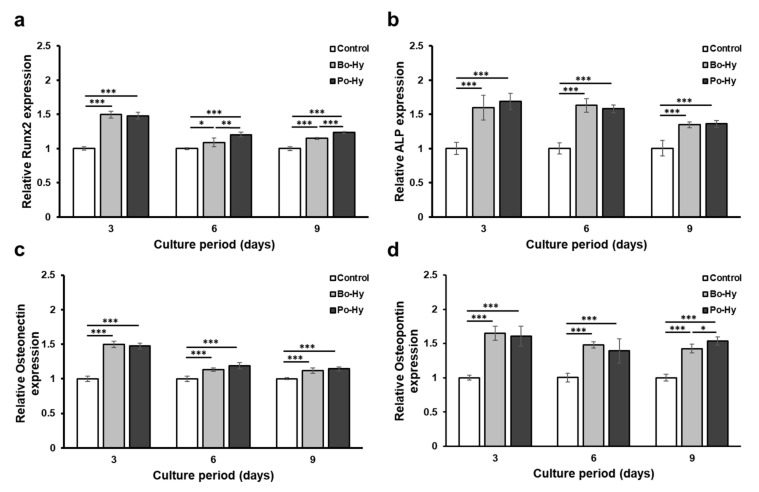
qPCR analysis of MC3T3-E1 cells on xenograft extracts. (**a**) Runt-related transcription factor 2 (Runx2), (**b**) ALP, (**c**) osteonectin (ON), (**d**) osteopontin (OPN) were selected as the osteogenic differentiation-related genes. Significant differences were observed when compared with the controls: * *p* < 0.05, ** *p* < 0.01, *** *p* < 0.001, *n* = 5.

To evaluate the effects of xenograft extracts on osteoblast differentiation, mRNA expression levels of Runx2, ALP, ON, and OPN were measured through qPCR. Compared with the control, the xenograft extracts upregulated the mRNA expression levels of Runx2, a key osteogenic transcriptional factor, and osteoblast markers, ALP, ON, and OPN (Figure 9). The Po-Hy and Bo-Hy extracts induced more mRNA expression of Runx2, ALP, ON, and OPN compared to the control. The mRNA expression of Runx2, which serves as a key regulator during osteoblast differentiation, was increased by approximately 50% at day 3 in both xenografts. In the cells treated with the xenograft extracts, the mRNA expression levels of ALP, ON, and OPN were also increased by 10–70% compared to the control, indicating that the xenografts have osteoinductivity.

### 3.2. In Vivo Findings

#### 3.2.1. Clinical Findings

Four rabbits recovered without any significant complications and postoperative healing proceeded statically. The xenografts placed in the defect areas of rabbits showed that newly formed bones were successfully infiltrating into the residual bone. There were no signs of damage or severe inflammation, necrosis, or osteolysis during the experimental period.

#### 3.2.2. Volumetric Findings

**Figure 10:**
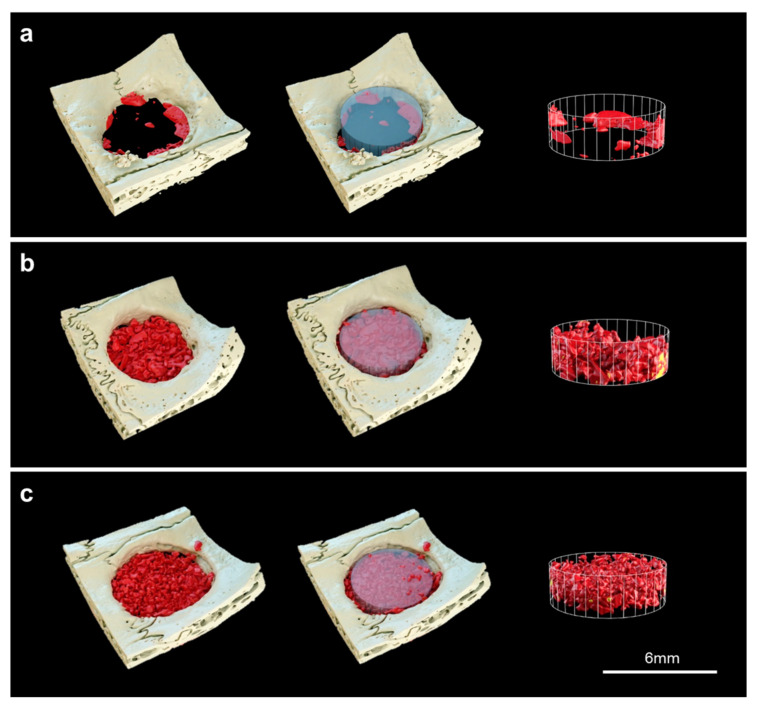
Reconstructed three-dimensional (3D) images within the 6 mm region of interest using micro-CT analysis after 8 weeks of healing. (**a**) Control, (**b**) Bo-Hy, and (**c**) Po-Hy (yellow: xenograft materials, red: newly formed bone, scale bars = 6 mm).

**Figure 11:**
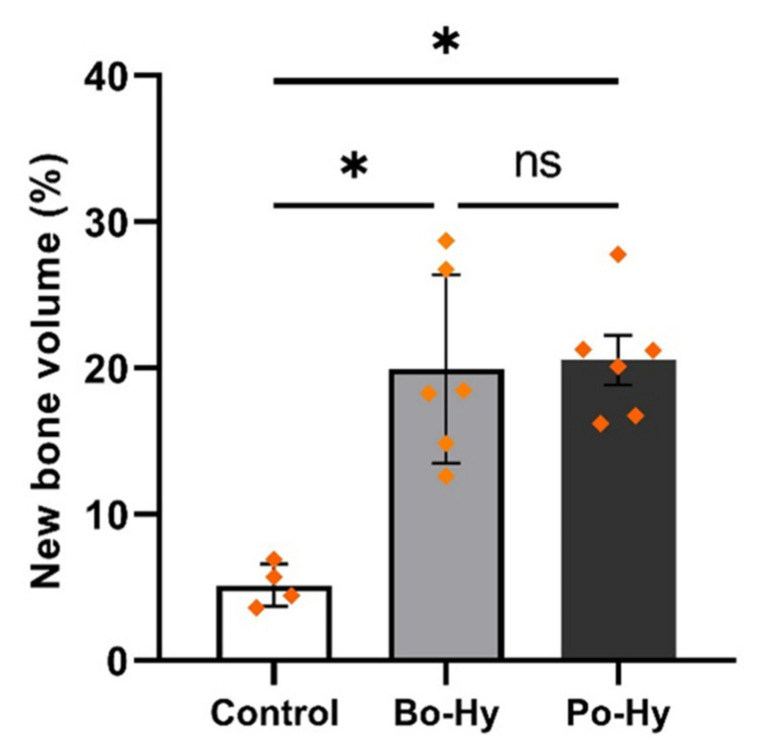
The percentage of new bone volume between the three groups determined by micro-CT analysis (ns: non-significant, *: *p* < 0.05). Differences between the two xenograft groups were not statistically significant.

New bone volume was observed of bone defects in micro-CT 3D images. Bo-Hy and Po-Hy groups were able to form bone with the highest bone density observed (Figure 10). Bone volume was significantly higher in the Bo-Hy and Po-Hy groups than the control at 8 weeks after surgery. Bo-Hy was 19.95 ± 6.45% and Po-Hy was 20.56 ± 4.16%. There was no significant difference between the Bo-Hy and Po-Hy groups (Figure 11, Table 3).

#### 3.2.3. Histologic Findings

**Figure 12:**
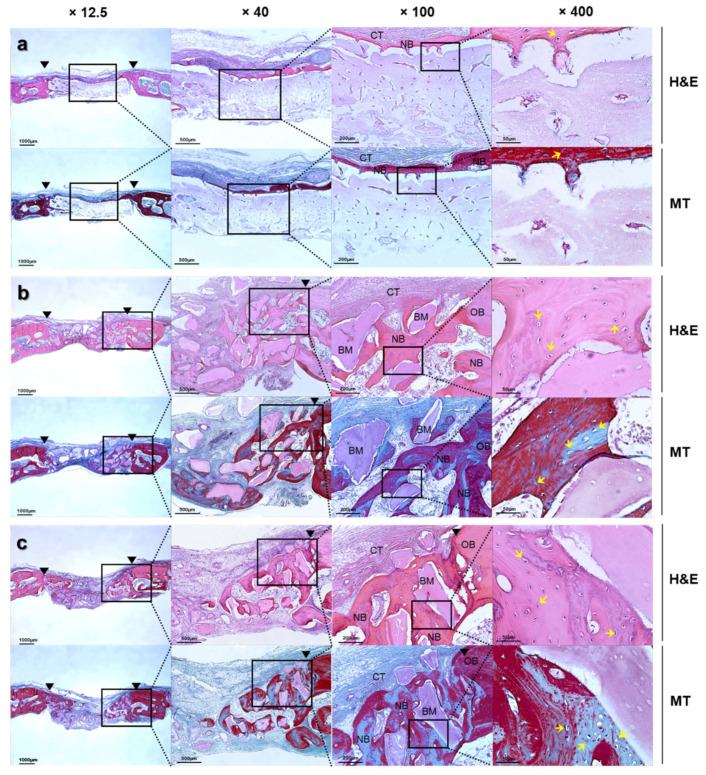
Histologic sections of calvaria defects from rabbits at 8 weeks after surgery. (**a**) Control, (**b**) Bo-Hy, and (**c**) Po-Hy (black arrow: defect border margin, yellow arrow: osteocyte, BM: bone graft material, NB: new bone, OB: old bone, CT: connective tissue, H&E: Hematoxylin & Eosin, MT: Masson’s trichrome).

The samples recovered after 8 weeks of implantation were histologically analyzed for neovascularization and new bone formation in the periphery and the interior of the defects by H&E and MT staining. The bone graft materials exhibited minimal resorption and maintained their shape despite the absence of a barrier. In H&E staining, inflammation cell-infiltrating, fibrotic, osteoclast, and osteoblast activity at the graft site was evaluated. In MT staining, blue indicates collagen fiber, and red indicates myofibrils, cytoplasm, or mature bone that appears inside the bone. As a result of H&E staining, no inflammation cells were found in all groups. In the two experimental groups, predominantly woven bones surrounded by osteoblasts around the bone graft material were observed. New bone maturation processes appeared with the mineralization of osteocytes in lacunae. In the MT staining results, the presence of mature bone formation surrounds the graft material from the defect boundary to the defect center and some areas of the mature bone (Figure 12).

#### 3.2.4. Histomorphometric Findings

**Figure 13:**
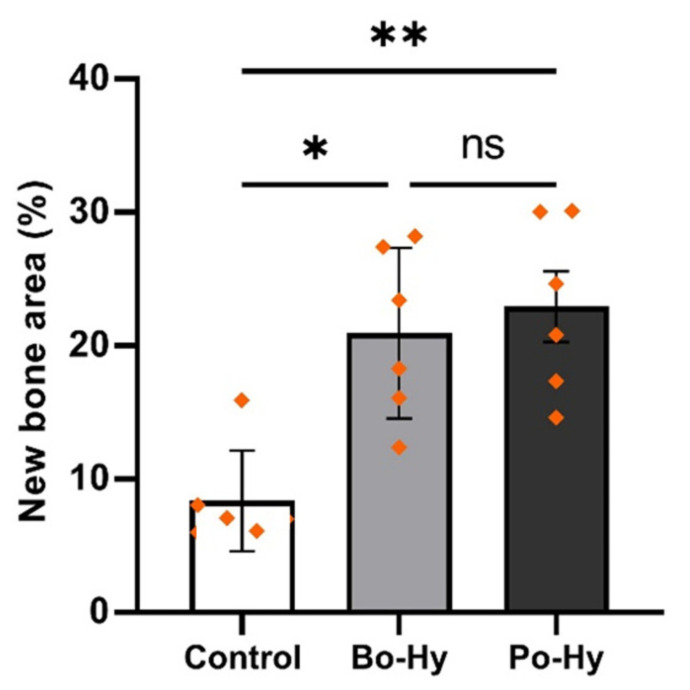
The percentage of new bone area determined by histological analysis (ns: non-significant, *: *p* < 0.05, **: *p* < 0.01). Differences between the two xenograft groups were not statistically significant.

The histological results at 8 weeks are shown in Figure 13 and Table 4. The predominantly woven bones surrounded by osteoblasts were observed in all groups at 8 weeks. The control group had the lowest bone formation at 8.37 ± 3.77%, whereas the Bo-Hy group was 20.97 ± 6.40% and the Po-Hy group had the highest bone formation at 22.94 ± 6.49%. The control group showed a statistically significant difference compared with the other groups (*p* < 0.05). However, there was no significant difference between Bo-Hy and Po-Hy groups.

## 4. Discussion

Hydroxypropyl methylcellulose (HPMC) is a hydrophilic carrier material used in drug delivery systems and dental devices [46]. The addition of HPMC to bone graft materials improves the moldability when in contact with water or biological fluids, maintaining the stability of the bone material. [47]. In this study, HPMC was added to bone graft materials to increase the moldability of particles and to facilitate application in bone defect sites.

Bovine-derived bone grafts were first to be used as xenografts in dentistry, and several bovine xenograft products are currently commercialized in the market for bone graft materials [48]. These grafts have an osteoconductive potential and are similar to the human bone in chemical and physical characteristics [49,50]. Recently, the porcine-derived xenograft has gained popularity as an alternative to the bovine-derived xenograft, and many products have been commercialized. Chang et al. [51] reported that porcine bones are composed of particles with an average size of 0.25–1.0 mm and a porosity as high as bovine bones. Bae et al. [38] suggested that a porcine xenograft had a non-inferior ability in new bone regeneration compared to that of a bovine xenograft. Therefore, the porcine-derived xenograft is an effective bone graft material for bone regeneration because it has high biocompatibility, excellent fusion ability to the graft site, and high bonding strength [52,53,54]. In this study, HPMC was mixed on the particle-type xenografts, which were derived from two different species (bovine and porcine), and bone formation ability was compared.

In a previous study, the osteogenic activity of porcine- and bovine-derived xenografts was evaluated using human mesenchymal stem cells, and both xenografts showed the similar osteoinductivity [38]. In this experiment, MC3T3-E1 cells, a pre-osteoblast derived from C57B/6 mice, were used to evaluate the osteoinductivity of the porcine and bovine xenografts with HPMC. Runx2, a major transcriptional regulator of osteoblast differentiation, regulates the expression of several osteogenic genes, including collagen I, ALP, OPN, bone sialoprotein, and bone calcium [55]. ALP, an early marker of osteoblast differentiation, is involved in bone mineralization [56]. ON and OPN, of the non-collagenous proteins abundant in the bone matrix, play important roles in bone formation [57,58]. Both xenografts showed an increase in cell proliferation and ALP activity, and induced more mRNA expression of Runx2, ALP, ON, and OPN compared with the controls. These results are similar to those of previous experiments and indicate that bovine- or porcine-derived xenografts with HPMC are suitable for use as bone substitutes [30].

A porous structure with various sizes of pores is essential for an ideal bone substitute [59]. The porosity and pore size play important roles in the efficacy of cell seeding, diffusion, and mechanical strength in the bone graft materials [60]. Porous bone graft materials mediate bone remodeling by assisting in vascularization, osseointegration from adjacent bones, and infiltration of osteoblasts and osteoclasts. In addition, the macropores contribute to increase the osteo-induction and the micropores contribute to enhance the osseointegration within bone graft substitutes [61]. In a previous study, the surface morphology of the bovine-derived xenograft with HPMC was shown as a macro-porous structure and without a distinct hydrogel layer [30]. In our study, the scanning electron microscopy (SEM) images of Bo-Hy and Po-Hy groups with HPMC showed that porous structures closely mimicked cancellous bone. Both groups are thought to provide osteoblast scaffolds as macro-porous structures appear. Furthermore, our surface investigation suggested that the hydrogel layer of HPMC was not visible, which concurred with previous studies [30].

From a surgical standpoint of the in vivo experiment, the Po-Hy group showed favorable handling properties similar to the Bo-Hy group and could be easily implanted into the bone defects. The two types of xenografts used in this study, with the inclusion of HPMC, increased the manipulability of the particles. They showed adhesion of the bone graft material to the bone defects. The experimental groups were HPMC-mixed particles, which by adding saline made it possible to be molded according to the size and desired shape of the defect and fixed in the bone defects. An additional barrier for the maintenance of space is unnecessary, which enables the cost reduction of the procedure.

In this study, a μCT analysis and histometric evaluations were conducted in a critical-sized rabbit calvaria defect to compare the bone-forming ability of bovine- and porcine-derived xenografts with HPMC. The newly formed bones surrounded by osteoblasts were distributed around the experimental xenografts in the Bo-Hy and Po-Hy groups. The experimental groups expressed superior bone cell proliferation and bone conduction compared to the control group. Both xenografts were observed to be maintained without structural collapse within the bone defect. Kim et al. [30] studied animal experiments using the bovine xenograft with HPMC and reported uniform new bone generation in rat calvaria at 8 weeks post-surgery. As a result of histomorphometric analysis, the area of new bone in the Bo-Hy group (20.97 ± 6.40%) and the Po-Hy group (22.94 ± 6.49%) had a larger new bone area than the control group (8.37 ± 1.25%) and showed a significant difference. In the comparison between the two species of xenografts with HPMC, the Po-Hy group did not show a significant difference compared to the Bo-Hy group. This indicated that the porcine xenograft with HPMC was not inferior in bone formation performance to the bovine xenograft with HPMC.

In this animal study, defects of 6 mm in diameter and 2 mm-thick were formed and the hydrogel xenografts were easily molded to fit the size of the bone defect. However, additional research on a large model with irregular defects should be performed to evaluate the manipulability of the bone graft material in consideration of the clinical situation. There is insufficient evidence on the appropriate mixing concentration of HPMC with the bone graft particles as the company did not expose the concentration of HPMC added to the two types of xenografts. Within the limits of this study, the surface properties, cell activity, and bone regeneration ability of the porcine xenograft with HPMC were similar to those of the bovine-derived xenograft with HPMC. Furthermore, the two types of HPMC-mixed xenografts are moldable and easily shaped to conform to bone defects. Therefore, xenografts with HPMC are suitable for bone grafting due to their increased manipulability during surgery, and the porcine-derived xenograft with HPMC can be used as a biomaterial for bone regeneration. Further large animal studies on xenografts suitable for irregular and extensive bone defects of this bone graft material are needed. Further large animal studies are required to evaluate whether this bone graft material is appropriate for irregular and extensive bone defects.

## 5. Conclusions

Within the limitations of the present study, the porcine-derived xenograft with HPMC showed a similar bone-forming ability to the bovine-derived xenograft with HPMC. In addition, the two types of xenografts mixed with HPMC revealed excellent manipulability and were well-located in the bone defect. Based on these results, this study showed that the porcine-derived xenograft with HPMC is a promising alternative to the bovine-derived xenograft for guided bone regeneration in clinical situations.

## Figures and Tables

**Table 1:** Primer sequences used for the real-time polymerase chain reaction (PCR) analysis.

Target Genes	Sequences	Ref.
GAPDH	F: 5′-ACCACAGTCCATGCCATCAC-3′ R: 5′-TCAAATCTGCAGCTTCAAGG-3′	[43]
Runt-related transcription factor 2 (Runx2)	F: 5′-AAGCTGCGGCAAGACAAG-3′ R: 5′-TCAAATCTGCAGCTTCAAGG-3′	[44]
ALP	F: 5′-AAACCCAGAACACAAGCATTCC-3′ R: 5′-TCCACCAGCAAGAAGAAGCC-3′	[44]
Osteonectin (ON)	F: 5′-CTTCCTGCTGCTCCCCTCTA-3′ R: 5′-AGCAACTTCAGTCTGCTGAGGC-3′	This study
Osteopontin (OPN)	F: 5′-GACGGCCGAGGTGATAGCTT-3′ R: 5′-CATGGCTGGTCTTCCCGTTGC-3′	[45]

**Table 2:** Elemental chemical compositions of the two xenograft composites using EDX (atomic %, mean ± SD).

Elements	Chemical Compositions (wt.%)	
	Bo-Hy	Po-Hy
C	11.89 ± 0.59	9.96 ± 1.62
O	47.45 ± 5.79	45.69 ± 3.56
P	12.87 ± 1.77	13.80 ± 1.01
Ca	27.78 ± 4.74	30.56 ± 4.13
Ca/P	2.16	2.22

**Table 3:** Micro-CT analysis results of new bone volume for all samples at 8 weeks.

	Group	Mean ± SD	*p*-Value ^1^
New bone volume (%)	Control	5.17 ± 1.45	0.006 *
	Bo-Hy	19.95 ± 6.45	
	Po-Hy	20.56 ± 4.16	

^1^ Results were analyzed using the Kruskal–Wallis test. * Indicates statistically significant differences (*p* < 0.05).

**Table 4:** Histology analysis results of new bone area for all samples at 8 weeks.

	Group	Mean ± SD	*p*-Value ^1^
New bone area (%)	Control	8.37 ± 3.77	0.016 *
	Bo-Hy	20.97 ± 6.40	
	Po-Hy	22.94 ± 6.49	

^1^ Results were analyzed using the Kruskal–Wallis test. * Indicates statistically significant differences (*p* < 0.05).

## Data Availability

The data presented in this study are available upon request from the corresponding author.

## References

[1] Zhao R., Yang R., Cooper P.R., Khurshid Z., Shavandi A., Ratnayake J. (2021). Bone grafts and substitutes in dentistry: A review of current trends and developments. Molecules.

[2] Doonquah L., Holmes P.J., Ranganathan L.K., Robertson H. (2021). Bone grafting for implant surgery. Oral Maxillofac. Surg. Clin..

[3] Athanasiou V.T., Papachristou D.J., Panagopoulos A., Saridis A., Scopa C.D., Megas P. (2009). Histological comparison of autograft, allograft-DBM, xenograft, and synthetic grafts in a trabecular bone defect: An experimental study in rabbits. Med. Sci. Monit..

[4] Jang K.Y., Lee J.H., Oh S.H., Ham B.D., Chung S.M., Lee J.K., Ku J.K. (2020). Bone graft materials for current implant dentistry. J. Dent. Res..

[5] Damlar I., Arpağ O.F., Tatli U., Altan A. (2017). Effects of Hypericum perforatum on the healing of xenografts: A histomorphometric study in rabbits. Br. J. Oral Maxillofac. Surg..

[6] Tovar N., Jimbo R., Gangolli R., Perez L., Manne L., Yoo D., Lorenzoni F., Witek L., Coelho P.G. (2014). Evaluation of bone response to various anorganic bovine bone xenografts: An experimental calvaria defect study. Int. J. Oral Maxillofac. Surg..

[7] Hallman M., Lundgren S., Sennerby L. (2001). Histologic analysis of clinical biopsies taken 6 months and 3 years after maxillary sinus floor augmentation with 80% bovine hydroxyapatite and 20% autogenous bone mixed with fibrin glue. Clin. Implant. Dent. Relat. Res..

[8] Poumarat G., Squire P. (1993). Comparison of mechanical properties of human, bovine bone and a new processed bone xenograft. Biomaterials.

[9] Sheikh Z., Hamdan N., Ikeda Y., Grynpas M., Ganss B., Glogauer M. (2017). Natural graft tissues and synthetic biomaterials for periodontal and alveolar bone reconstructive applications: A review. Biomater. Res..

[10] Wong R.W.K., Rabie A.B.M. (2010). Effect of bio-oss^®^ collagen and collagen matrix on bone formation. Open Biomed. Eng. J..

[11] Park S.A., Shin J.W., Yang Y.I., Kim Y.K., Park K.D., Lee J.W., Jo I.H., Kim Y.J. (2014). In vitro study of osteogenic differentiation of bone marrow stromal cells on heat-treated porcine trabecular bone blocks. Biomaterials.

[12] Bracey D.N., Seyler T.M., Jinnah A.H., Lively M.O., Willey J.S., Smith T.L., Dyke M.E.V., Whitlock P.W. (2018). A decellularized porcine xenograft-derived bone scaffold for clinical use as a bone graft substitute: A critical evaluation of processing and structure. J. Funct. Biomater..

[13] Valencia-Llano C.H., López-Tenorio D., Grande-Tovar C.D. (2022). Biocompatibility Assessment of Two Commercial Bone Xenografts by In Vitro and In Vivo Methods. Polymers.

[14] Le B.T., Borzabadi-Farahani A. (2014). Simultaneous implant placement and bone grafting with particulate mineralized allograft in sites with buccal wall defects, a three-year follow-up and review of literature. J. Craniomaxillofac. Surg..

[15] Seo Y.H., Hwang S.H., Kim Y.N., Kim H.J., Bae E.B., Huh J.B. (2022). Bone Reconstruction Using Two-Layer Porcine-Derived Bone Scaffold Composed of Cortical and Cancellous Bones in a Rabbit Calvarial Defect Model. Int. J. Mol. Sci..

[16] Jung G.U., Hong S.J., Hong J.Y., Pang E.K. (2016). Histomorphometric evaluation of onlay freeze-dried block bone and deproteinized bovine bone with collagen in rat. Tissue Eng. Regen. Med..

[17] Gehrke S.A., Mazón P., Del Fabbro M., Tumedei M., Aramburú Júnior J., Pérez-Díaz L., De Aza P.N. (2019). Histological and histomorphometric analyses of two bovine bone blocks implanted in rabbit calvaria. Symmetry.

[18] Hwang K.S., Choi J.W., Kim J.H., Chung H.Y., Jin S., Shim J.H., Yun W.S., Jeong C.M., Huh J.B. (2017). Comparative efficacies of collagen-based 3D printed PCL/PLGA/β-TCP composite block bone grafts and biphasic calcium phosphate bone substitute for bone regeneration. Materials.

[19] Yoo H.S., Bae J.H., Kim S.E., Bae E.B., Kim S.Y., Choi K.H., Moon K.O., Jeon C.M., Huh J.B. (2017). The effect of bisphasic calcium phosphate block bone graft materials with polysaccharides on bone regeneration. Materials.

[20] Kim Y.K., Ku J.K. (2020). Ridge augmentation in implant dentistry. J. Korean Assoc. Oral Maxillofac. Surg..

[21] Wei W., Ma Y., Yao X., Zhou W., Wang X., Li C., Lin J., He Q., Leptihn S., Ouyang H. (2021). Advanced hydrogels for the repair of cartilage defects and regeneration. Bioact. Mater..

[22] Mukherjee I. (2022). Recent Development of Polysaccharide-Derived Hydrogel: Properties, Stimuli-Responsiveness and Bioapplications. Polym. Sci..

[23] Mahaguna V., Talbert R.L., Peters J.I., Adams S., Reynolds T.D., Lam F.Y., Williams III R.O. (2003). Influence of hydroxypropyl methylcellulose polymer on in vitro and in vivo performance of controlled release tablets containing alprazolam. Eur. J. Pharm. Biopharm..

[24] Tozzi G., De Mori A., Oliveira A., Roldo M. (2016). Composite hydrogels for bone regeneration. Materials.

[25] Liu W., Zhang J., Weiss P., Tancret F., Bouler J.M. (2013). The influence of different cellulose ethers on both the handling and mechanical properties of calcium phosphate cements for bone substitution. Acta. Biomater..

[26] Chang C., Zhang L. (2011). Cellulose-based hydrogels: Present status and application prospects. Carbohydr. Polym..

[27] Zhang J., Liu W., Gauthier O., Sourice S., Pilet P., Réthoré G., Khairoun K., Bouler J.M., Tancret F., Weiss P. (2016). A simple and effective approach to prepare injectable macroporous calcium phosphate cement for bone repair: Syringe-foaming using a viscous hydrophilic polymeric solution. Acta. Biomater..

[28] Chen I.C., Su C.Y., Lai C.C., Tsou Y.S., Zheng Y., Fang H.W. (2021). Preparation and characterization of moldable demineralized bone matrix/calcium sulfate composite bone graft materials. J. Funct. Biomater..

[29] Chen C., Xi Y., Weng Y. (2022). Recent Advances in Cellulose-Based Hydrogels for Tissue Engineering Applications. Polymers.

[30] Kim S.Y., Lee Y.J., Cho W.T., Hwang S.H., Heo S.C., Kim H.J., Huh J.B. (2021). Preliminary Animal Study on Bone Formation Ability of Commercialized Particle-Type Bone Graft with Increased Operability by Hydrogel. Materials.

[31] Feroz S., Dias G. (2021). Hydroxypropylmethyl cellulose (HPMC) crosslinked keratin/hydroxyapatite (HA) scaffold fabrication, characterization and in vitro biocompatibility assessment as a bone graft for alveolar bone regeneration. Heliyon.

[32] Urban R.M., Turner T.M., Hall D.J., Infanger S.I., Cheema N., Lim T.H., Richelsoph K. (2004). An injectable calcium sulfate-based bone graft putty using hydroxypropylmethylcellulose as the plasticizer. Orthopedics.

[33] Gauthier O., Müller R., von Stechow D., Lamy B., Weiss P., Bouler J.M., Aguado E., Daculsi G. (2005). In vivo bone regeneration with injectable calcium phosphate biomaterial: A three-dimensional micro-computed tomographic, biomechanical and SEM study. Biomaterials.

[34] Gibbs D.M., Black C.R., Dawson J.I., Oreffo R.O. (2016). A review of hydrogel use in fracture healing and bone regeneration. J. Tissue Eng. Regen. Med..

[35] Ferreira N.N., Ferreira L.M.B., Cardoso V.M.O., Boni F.I., Souza A.L.R., Gremião M.P.D. (2018). Recent advances in smart hydrogels for biomedical applications: From self-assembly to functional approaches. Eur. Polym. J..

[36] Datta P., Dhara S., Chatterjee J. (2012). Hydrogels and electrospun nanofibrous scaffolds of N-methylene phosphonic chitosan as bioinspired osteoconductive materials for bone grafting. Carbohydr. Polym..

[37] Salamanca E., Hsu C.C., Huang H.M., Teng N.C., Lin C.T., Pan Y.H., Chang W.J. (2018). Bone regeneration using a porcine bone substitute collagen composite in vitro and in vivo. Sci. Rep..

[38] Bae E.B., Kim H.J., Ahn J.J., Bae H.Y., Kim H.J., Huh J.B. (2019). Comparison of bone regeneration between porcine-derived and bovine-derived xenografts in rat calvarial defects: A non-inferiority study. Materials.

[39] Kim J.W., Shin Y.C., Lee J.J., Bae E.B., Jeon Y.C., Jeong C.M., Yun M.J., Lee S.H., Han D.W., Huh J.B. (2017). The Effect of Reduced Graphene Oxide-Coated Biphasic Calcium Phosphate Bone Graft Material on Osteogenesis. Int. J. Mol. Sci..

[40] Cai L., Zhang J., Qian J., Li Q., Li H., Yan Y., Wei S., Wei J., Su J. (2018). The effects of surface bioactivity and sustained-release of genistein from a mesoporous magnesium-calcium-silicate/PK composite stimulating cell responses in vitro, and promoting osteogenesis and enhancing osseointegration in vivo. Biomater. Sci..

[41] Park J.C., Bae E.B., Kim S.E., Kim S.Y., Choi K.H., Choi J.W., Bae J.H., Ryu J.J., Huh J.B. (2016). Effects of BMP-2 Delivery in Calcium Phosphate Bone Graft Materials with Different Compositions on Bone Regeneration. Materials.

[42] Moon K., Lee S., Cha J. (2020). Bacillus subtilis fermentation of Malva verticillata leaves enhances antioxidant activity and osteoblast differentiation. Foods.

[43] Sakisaka Y., Kanaya S., Nakamura T., Tamura M., Shimauchi H., Nemoto E. (2016). p38 MAP kinase is required for Wnt3a-mediated osterix expression independently of Wnt-LRP5/6-GSK3β signaling axis in dental follicle cells. Biochem. Biophys. Res. Commun..

[44] Kang H.R., Yun H.S., Lee T.K., Lee S., Kim S.H., Moon E., Park K.M., Kim K.H. (2018). Chemical characterization of novel natural products from the roots of asian rice (Oryza sativa) that control adipocyte and osteoblast differentiation. J. Agric. Food Chem..

[45] Qing W., Guang-Xing C., Lin G., Liu Y. (2012). The osteogenic study of tissue engineering bone with BMP2 and BMP7 gene-modified rat adipose-derived stem cell. J. Biotechnol. Biomed..

[46] Siepmann J., Peppas N.A. (2012). Modeling of drug release from delivery systems based on hydroxypropyl methylcellulose (HPMC). Adv. Drug Deliv. Rev..

[47] Sadiasa A., Sarkar S.K., Franco R.A., Min Y.K., Lee B.T. (2014). Bioactive glass incorporation in calcium phosphate cement-based injectable bone substitute for improved in vitro biocompatibility and in vivo bone regeneration. J. Biomater. Appl..

[48] Lee J.H., Yi G.S., Lee J.W., Kim D.J. (2017). Physicochemical characterization of porcine bone-derived grafting material and comparison with bovine xenografts for dental applications. J. Periodontal. Implant. Sci..

[49] Pripatnanont P., Nuntanaranont T., Vongvatcharanon S. (2019). Proportion of deproteinized bovine bone and autogenous bone affects bone formation in the treatment of calvarial defects in rabbits. Int. J. Oral Maxillofac. Surg..

[50] Fernandes Y., Mantovani R., Reino D., Novaes Jr A., Messora M., Gustavo Sousa L., Palioto D., Scombatti de Souza S. (2022). Evaluation of a New Porcine Bone Graft on the Repair of Surgically Created Critical Bone Defects in Rat Calvaria: Histomorphometric and Microtomographic Study. J. Funct. Biomater..

[51] Chang L.C. (2021). Comparison of Clinical Parameters in Dental Implant Therapy between Implant Site Development Using Porcine-and Bovine-Derived Xenografts. Technologies.

[52] Lee J.S., Shin H.K., Yun J.H., Cho K.S. (2017). Randomized clinical trial of maxillary sinus grafting using deproteinized porcine and bovine bone mineral. Clin. Implant. Dent. Relat. Res..

[53] Guarnieri R., DeVilliers P., Grande M., Stefanelli L.V., Di Carlo S., Pompa G. (2017). Histologic evaluation of bone healing of adjacent alveolar sockets grafted with bovine-and porcine-derived bone: A comparative case report in humans. Regen. Biomater..

[54] Lai V.J., Michalek J.E., Liu Q., Mealey B.L. (2020). Ridge preservation following tooth extraction using bovine xenograft compared with porcine xenograft: A randomized controlled clinical trial. J. Periodontol..

[55] Ducy P. (2000). Cbfa1: A molecular switch in osteoblast biology. Dev. Dyn..

[56] Golub E.E., Boesze-Battaglia K. (2007). The role of alkaline phosphatase in mineralization. Curr. Opin. Orthop..

[57] Zhu Y.S., Gu Y., Jiang C., Chen L. (2020). Osteonectin regulates the extracellular matrix mineralization of osteoblasts through P38 signaling pathway. J. Cell. Physiol..

[58] Morinobu M., Ishijima M., Rittling S.R., Tsuji K., Yamamoto H., Nifuji A., Denhardt D., Noda M. (2003). Osteopontin expression in osteoblasts and osteocytes during bone formation under mechanical stress in the calvarial suture in vivo. J. Bone Miner. Res..

[59] LeGeros R.Z. (1991). Calcium phosphate in Oral Biology and Medicine. Monogr. Oral Sci..

[60] Bertoldi S., Farè S., Tanzi M.C. (2011). Assessment of scaffold porosity: The new route of micro-CT. J. Appl. Biomater. Biomech..

[61] Petrochenko P., Narayan R.J. (2010). Novel approaches to bone grafting: Porosity, bone morphogenetic proteins, stem cells, and the periosteum. J. Long-Term Eff. Med. Implant..

